# Development of a Stringent Ex Vivo-Burned Porcine Skin Wound Model to Screen Topical Antimicrobial Agents

**DOI:** 10.3390/antibiotics13121159

**Published:** 2024-12-02

**Authors:** Ping Chen, Eliza A. Sebastian, S. L. Rajasekhar Karna, Kai P. Leung

**Affiliations:** Combat Wound Care Group, CRT 4, United States Army Institute of Surgical Research, JBSA Fort Sam Houston, San Antonio, TX 78234, USA; ping.chen.ctr@health.mil (P.C.); eliza.a.sebastian.ctr@health.mil (E.A.S.); sailakshmirajasekh.karna.ctr@health.mil (S.L.R.K.)

**Keywords:** ex vivo, model, burn, stringent, antimicrobial, commensal, Silvadene, Flammacerium, *Pseudomonas*, *Staphylococcus*

## Abstract

**Background**: Due to rising antibiotic-resistant microorganisms, there is a pressing need to screen approved drugs for repurposing and to develop new antibiotics for controlling infections. Current in vitro and ex vivo models have mostly been unsuccessful in establishing in vivo relevance. In this study, we developed a stringent ex vivo-burned porcine skin model with high in vivo relevance to screen topical antimicrobials. **Methods**: A 3 cm-diameter thermal injury was created on non-sterilized porcine skin using a pressure-monitored and temperature-controlled burn device. Commensals were determined pre- and post-burn. The burn wound was inoculated with a target pathogen, and efficacies of Silvadene, Flammacerium, Sulfamylon, and Mupirocin were determined. The in vivo relevance of this platform was evaluated by comparing the ex vivo treatment effects to available in vivo treatment outcomes (from our laboratory and published reports) against selective burn pathogens. **Results**: Approximately 1% of the commensals survived the skin burn, and these commensals in the burn wounds affected the treatment outcomes in the ex vivo screening platform. When tested against six pathogens, both Silvadene and Flammacerium treatment exhibited ~1–3 log reduction in viable counts. Sulfamylon and Mupirocin exhibited higher efficacy than both Silvadene and Flammacerium against *Pseudomonas* and *Staphylococcus,* respectively. The ex vivo treatment outcomes of Silvadene and Flammacerium against *Pseudomonas* were highly comparable to the outcomes of the in vivo (rats). **Conclusions**: The ex vivo model developed in our lab is a stringent and effective platform for antimicrobial activity screening. The outcome obtained from this ex vivo model is highly relevant to in vivo.

## 1. Introduction

Wound infection is highly correlated to mortality and morbidity in burn patients and is responsible for up to 50% of burn-related deaths [1]. To control the wound infection, systemic antibiotic, topical antibiotic, or combination (systemic plus topical) therapy is the common practice [2]. However, infected burn patients treated with various antimicrobial regimens are at higher risk of developing more serious infections from multi-drug-resistant organisms. For example, multidrug-resistant gram-negative bacteria *Acinetobacter baumannii*, *Klebsiella pneumoniae*, *Pseudomonas aeruginosa*, and *Proteus mirabilis* are commonly identified in burn wound infections [3]. Bacterial resistance has been detected in nearly all antibiotics currently in clinical use, and there are only a few novel drugs in the pipeline to combat drug-resistant microorganisms, especially drug-resistant gram-negative bacteria [4]. Additionally, global warming could make the resistance worse. As the globe warms, frequent high temperatures increase bacterial growth rates [5] and horizontal gene transfer [6], which contributes to the rise in antimicrobial resistance occurrences [7]. Therefore, the increased resistance of bacteria to antibiotics and other antimicrobials is a global health threat that makes infections harder to treat and may exacerbate overall infection, causing increased healthcare costs, morbidity, and mortality [8,9,10]. Due to the huge global health threat posed by these antibiotic-resistant microorganisms, there is a pressing need for repurposing screening of approved drugs and developing new antibacterial drugs to treat various wound infections better.

Within the preclinical drug development pipeline, in vitro screening is commonly used in drug repurposing and discovery. Compared to in vivo methods, in vitro methods have multiple advantages, such as low cost, simplicity, tightly controlled, high throughput, and fewer ethical considerations [11]. However, in vitro models are limited in their ability to recapitulate host pathophysiology, complex interactions among the host immune system, pathogenicity of infectious agents, tissue heterogeneity, wound location, and severity of the complex clinical features of various injuries [12,13]. As a result, the outcomes from in vitro settings could be significantly different from the in vivo outcomes. To our best understanding, there are no reports available regarding the correlation between the in vitro and in vivo screening outcomes.

Animal models, which include mouse, rat, pig, rabbit, dog, sheep, and goat, can address the major shortcomings of in vitro studies and have become the gold standard for experimental studies related to external injuries [12,13]. However, it is hard to justify using animals as an initial screening platform for a large number of candidate therapeutics due to high costs and ethical concerns. Therefore, there is a need for more cost-effective screening methods that could generate outcomes with high in vivo relevance.

Ex vivo models using animal tissues largely retain the tissue heterogeneity, architecture, and wound bed components, providing a more realistic matrix with a tightly controlled environment for studying wound infection and treatment response. To bridge the gap between in vitro assays and animal studies, several ex vivo models have been developed [14,15,16,17,18,19,20] for burn and infection control. Each model has its own merits and limitations. However, there are two noticeable drawbacks for the current ex vivo models: a small burn size and the disinfection of the surface before the induction of the burns. The diameters of the burns created in these models are less than 10 mm. Clinically, burn wound size and depth influence treatment response. For instance, a higher mortality rate is associated with a higher total burn surface area [21]. The small burn size could limit the biomass of pathogens, potentially resulting in a false positive treatment effect of the compound(s) tested. Additionally, the surface disinfection using disinfectants such as hydrogen peroxide, ethanol, and sodium hypochlorite [19] would significantly reduce (if not eliminate) the commensals and, consequently, the overall bacterial burden. In real situations, surface disinfection of the skin before a burn injury does not occur. Additionally, the antibiotic treatment effect against pathogens varies between single-species and multi-species infections [22]. It has been reported that the susceptibility to antibiotics could be altered by interspecies interactions by different potential mechanisms, such as target degradation [23,24], changing the dynamics of the pathogen cell density [22], inhibiting the colonization of the pathogen [25], and the acquisition of resistance element(s) from commensals [26]. The lack of multi-species interactions among the inoculated pathogen(s) and the absence of commensals due to disinfection in these ex vivo models could overestimate the treatment outcomes and in vivo relevance. Furthermore, to our knowledge, no report is available regarding the correlation (if any) between the screening outcome(s) using the existing ex vivo models and the results obtained from in vivo models.

Here, we report a stringent ex vivo model without using skin disinfection to take into consideration the commensals and use a larger 3 cm burned porcine wound for screening antibacterial formulations against wound pathogens. The results of this study, when compared to the in vivo treatment outcomes from either our laboratory or the literature, indicate that the stringent ex vivo model is sufficient to evaluate the efficacy of anti-bacterial formulations and is highly comparable to in vivo outcomes.

## 2. Results

### 2.1. Skin Commensals Are Present Pre- and Post-Skin Burn

**Figure 1:**
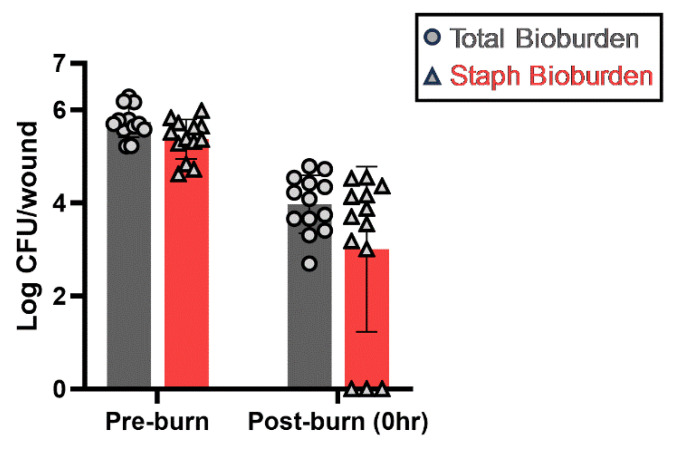
Total and staphylococci commensal bacteria on the commercial porcine skins before and after the burn. The bacterial numbers were transformed into logs and plotted as mean ± standard deviation. *n* ≥ 12 wounds/group, with a minimum of three biological repeats. Circles and triangles represent the total commensal bacterial number and staphylococci number, respectively.

To determine the presence of commensals on the skin, 3 cm-diameter skins were cut out from different batches of commercially available frozen porcine skins obtained from different animals. The skins were homogenized, serially diluted, and plated on 5% sheep blood Tryptic Soy Agar (TSA) plates and CHROMagar^TM^ Staph aureus selective plates. As shown in Figure 1, there were approximately 5.73 ± 0.33 log of viable commensals per 3 cm diameter of skin before the burn. The majority (about 5.37 log) of these commensals were staphylococci. After a 15 s burn, the number of commensal bacteria decreased by 1.9 log to approximately 3.97 ± 0.62 log per 3 cm diameter of skin. Among those that survived, close to 3 log of the commensal bacteria were staphylococci.

### 2.2. Skin Disinfection with Betadine Before Burns Enhanced Treatment Effect Against Pseudomonas aeruginosa Infection

As skin commensals and their diversity, wound environment, and presence of pathogens could influence wound infection, we aimed to understand if surface disinfection (reduction in microbial load) could influence the effectiveness of antimicrobial treatment. Therefore, we investigated the treatment outcomes of two antimicrobial creams, Silvadene and Flammacerium, against the growth of *P. aeruginosa* on surface-disinfected vs. non-disinfected burned skins.

**Figure 2:**
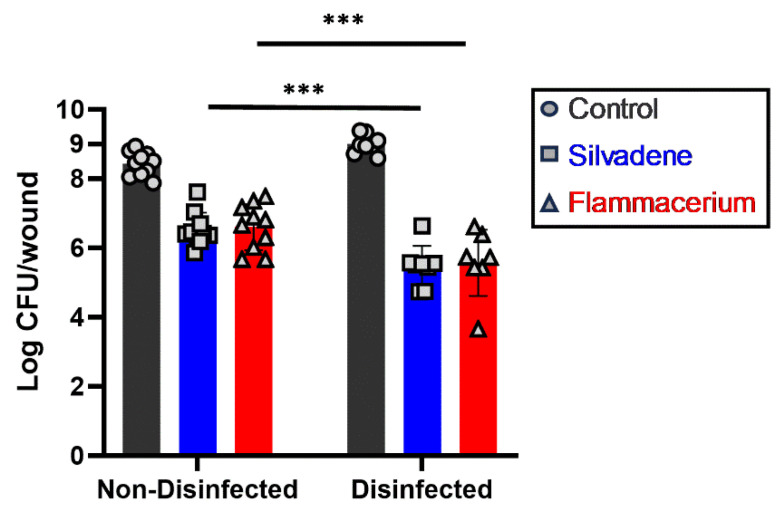
Disinfecting the skin surface with betadine before the burn increased the treatment effect. *P. aeruginosa* was grown on both disinfected and non-disinfected porcine skin in the presence or absence of treatment with antimicrobial creams, Silvadene and Flammacerium. The bacterial numbers were transformed into logs. *n* ≥ 12 wounds/group, with a minimum of three biological repeats. Significant differences were based on two-way ANOVA analyses followed by Tukey’s multiple comparison test. *** *p* < 0.001. Each bar represents mean ± standard deviation.

As shown in Figure 2, both surface-disinfected and non-disinfected 3 cm-diameter burned skins were infected with *P. aeruginosa* (approximately 2 × 10^4^ colony forming unit, CFU, per burn wound). Without treatment, *P. aeruginosa* grew approximately half a log more in the disinfected burn wounds than in the non-disinfected wounds prior to the burns. Furthermore, the growth of *P. aeruginosa* on surface-disinfected burned skin was significantly more susceptible to Silvadene and Flammacerium treatment, resulting in a 3.5 and 3.4 log reduction compared to their untreated control, respectively. In contrast, the growth of *P. aeruginosa* on non-disinfected skins was less susceptible to Silvadene and Flammacerium treatment, resulting in a 1.9 and 1.8 log reduction compared to the untreated control, respectively.

### 2.3. Stringent Ex Vivo Model Has High In Vivo Relevance (Rats) as Compared to Conventional Ex Vivo Model Based on Treatment Efficacy Outcomes Against P. aeruginosa Infection

**Figure 3:**
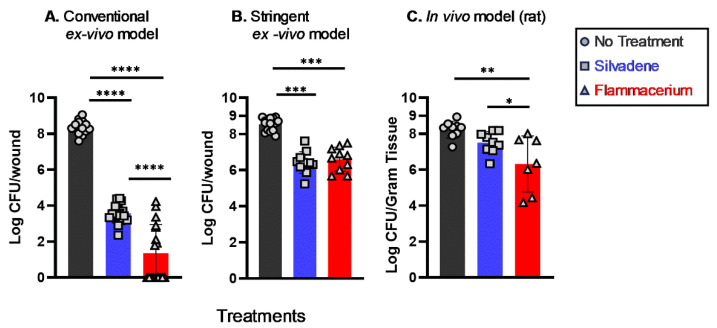
Treatment effects observed in our stringent ex vivo burn wound infection model were comparable to in vivo studies. Treatment outcomes of Silvadene and Flammacerium against *P. aeruginosa* were compared among the conventional ex vivo burn wound infection model (**A**), our stringent ex vivo burn wound infection model (**B**), and an in vivo (rat) model of burn wound infection (**C**). For both the conventional and stringent ex vivo methods, there were *n* ≥ 10 wounds per group, with three or more biological replicates, and for the in vivo study, *n* ≥ 7 rats per group. Significant differences were based on one-way ANOVA analyses followed by Tukey’s multiple comparison post-test. * *p* < 0.05, ** *p* < 0.01, *** *p* < 0.001 and **** *p* < 0.0001.

To determine which model mirrors in vivo treatment outcomes, we compared the conventional ex vivo model and our model (stringent ex vivo model) outcomes to the in vivo model of burn wound infection. In the conventional ex vivo burn wound infection model (Figure 3A), topical Silvadene treatment reduced the *Pseudomonas* viable count by about 4.8 log (from 8.4 log in control to 3.6 log), while topical Flammacerium treatment resulted in approximately 7 log reduction (from 8.4 log to 1.4 log) of *Pseudomonas* viable count.

In our ex vivo model (Figure 3B), topical Silvadene treatment reduced the *Pseudomonas* viable counts by about 2.1 log (from 8.5 log in control to 6.4 log), while topical Flammacerium treatment resulted in approximately 1.9 log reduction (from 8.5 log to 6.6 log) of *Pseudomonas* viable counts.

In the in vivo (rat) model (Figure 3C), Silvadene and Flammacerium treatment reduced the *Pseudomonas* viable counts by 0.7 log (8.2 to 7.5) and 1.6 log (8.2 to 6.3), respectively. By comparing the ex vivo outcomes to the in vivo outcomes, it is apparent that the conventional ex vivo model of burn wound infection overestimated the antimicrobial efficacy of topical antimicrobial formulations, while our ex vivo model outcomes aligned with the in vivo results.

### 2.4. Efficacy of Topical Antimicrobial Creams Against Commonly Encountered Burn Wound Pathogens in a Stringent Ex Vivo Model Burn Wound Infection

**Figure 4:**
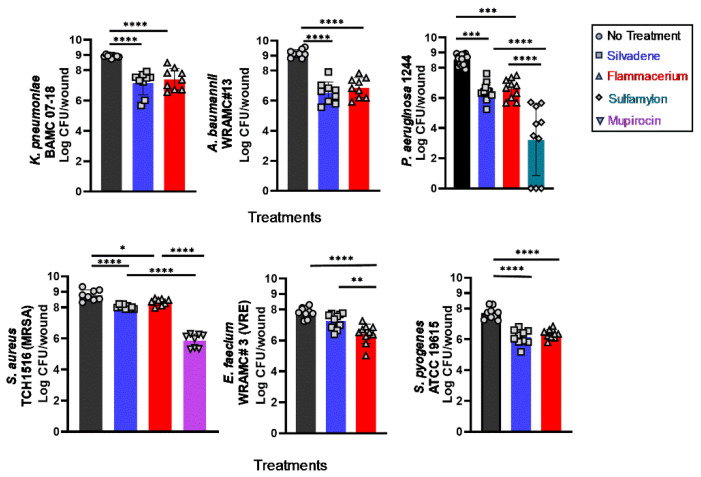
Antimicrobial efficacy of commonly used topical antimicrobial creams against frequently encountered burn wound pathogens. *n* ≥ 10 wounds/group, with a minimum of three biological repeats. Significant differences were based on one-way ANOVA analyses followed by Tukey’s multiple comparison post-test. * *p* < 0.05, ** *p* < 0.01, *** *p* < 0.001 and **** *p* < 0.0001. Each bar represents mean ± standard deviation.

Using our ex vivo burn wound infection model, we screened topical Silvadene and Flammacerium against several commonly encountered burn wound pathogens. These wound pathogens included multidrug-resistant clinical isolates of *Klebsiella pneumoniae* BAMC 07-18, *Acinetobacter baumannii* WRAMC#13, *Enterococcus faecium* WRAMC#3, *Staphylococcus aureus* TCH1516 (MRSA), and *Streptococcus pyogenes* ATCC 19615. As shown in Figure 4, all tested pathogens grew well on the non-disinfected ex vivo burned skins.

Silvadene and Flammacerium exhibited similar efficacy against multi-drug resistant *K*. *pneumoniae*, *A. baumannii*, and *S. pyogenes*. When tested against *Klebsiella*, Silvadene and Flammacerium treatment resulted in approximately 1.7 log (from 8.9 log in control to 7.2 log) and 1.5 log reductions (from 8.9 log to 7.4 log) in viable counts, respectively. 

Against *Acinetobacter*, Silvadene and Flammacerium treatments resulted in approximately 2.6 and 2.3 log reduction in viable counts, respectively. Topical Sulfamylon^®^ (mafenide acetate cream) exhibited a significantly stronger antimicrobial effect against *Pseudomonas* than that of Silvadene and Flammacerium, resulting in about a 6.3 log reduction in the *Pseudomonas* viable counts.

Mupirocin exhibited a stronger antimicrobial effect against *S. aureus* TCH1516 (MRSA) than that of Silvadene and Flammacerium. Mupirocin treatment reduced *S. aureus* viable counts by approximately 3 log, while both the Silvadene and Flammacerium treatments resulted in less than one log reduction in *S. aureus* viable counts.

Silvadene treatment reduced the *Enterococcus* burden by about 0.5 log (from 7.8 log in control to 7.3 log), while Flammacerium treatment resulted in approximately 1.4 log reduction (from 7.8 log to 6.4 log). Flammacerium exhibited approximately 1 log higher efficacy than Silvadene at reducing viable counts of *E. faecium.* Against *S. pyogenes*, Silvadene treatment reduced the viable counts by about 1.6 log (from 7.7 log in control to 6.1 log), while Flammacerium treatment resulted in approximately 1.4 log reduction (from 7.7 log to 6.3 log) in viable counts.

## 3. Discussion

Incidences of microbial resistance have been steadily rising over recent decades, resulting in increased healthcare costs, morbidity, and mortality [9,10]. There is an urgent need for the development of innovative treatments and new antimicrobials. Because the development process is extremely costly and has a low success rate, investment in antimicrobial discovery is not attractive to pharmaceutical companies and new drug development has been stagnant. One major hurdle of new drug development is the poor correlation between promising in vitro outcomes and in vivo efficacy before entering clinical trials. The high cost and ethical issues associated with preclinical animal studies make it more challenging to perform screening and down-select promising agent(s) in vivo. If the lead optimization screenings can be performed by a “fail-fast-and-fail-cheap” approach before the animal verification step, it would make antimicrobial drug discovery and subsequent commercialization more approachable.

In this study, we described a stringent ex vivo model to bridge the gap between the in vitro and in vivo models for screening topical antimicrobial agents and formulations. By comparing the treatment outcomes of different antimicrobial agents and formulations against commonly encountered burn wound pathogens, we showed that our stringent ex vivo approach produced results that had high in vivo relevance.

### 3.1. Our Ex Vivo Model Is Stringent

In clinics, the treatment outcomes could be influenced by multiple factors, such as burn size, burn depth, microbial load, microbial diversity, and the presence of wound pathogens. The burn wound sizes of ex vivo models reported in the literature varied but were always less than 10 mm in diameter. For example, a burn wound size of 4.8 mm was used in De Maesschalck’s study [16], 5 mm burn wounds were used in the model developed by Aves’ group [14], 8 mm burn wounds were used in Anderson’s study [15], and 2.9 mm burn wounds were used in the high-throughput platform study reported by Melnikov’s group [20]. Small burn wounds have a high margin of error in inoculation and bacterial counts. Hence, in our model, we increased the burn wound size to a 3 cm diameter to reduce such errors.

To mirror burn injuries in real life, we did not disinfect the skin surface before burns. This is contrary to the ex vivo model of burn wound infection reported in the literature in which the skins were disinfected before burns. Therefore, in our model, there were commensals that could interact with inoculated pathogen(s) as in the actual burn wounds. Close to 4 log CFU commensals per wound remained after skin burns. The survived commensals could interact with test pathogen(s) and potentially could alter the treatment outcomes. It has been shown that the presence of other bacteria could alter the susceptibility of a pathogen to antibiotic(s) [27,28]. Similarly, the virulence and antibiotic resistance of a pathogen could be modulated by multiple factors, such as the presence of commensals [29]. Additionally, the response of a pathogen to clinically relevant antibiotics could be altered by the interspecies interactions occurring in the microbiota [30]. Furthermore, mixed-species communities can facilitate the spread of antibiotic resistance, resulting in cross-protection against multiple antibacterial agents [31,32]. Therefore, rather than against a stand-alone pathogen as in the conventional ex vivo models, our stringent ex vivo model targeted test pathogens together with the commensals consisting of a mixed flora, a scenario that is comparable to the in vivo conditions of acute wounds.

Several parameters, such as burn temperature, burn duration, the surface area of the burn, and pressure applied by the burn device (for contact burns), have a significant effect on generating reproducible burns and, therefore, the final results [33]. To produce consistent burn size and depth, our burn device had a pressure gauge to monitor the applied pressure and an Advanced Multiparameter controller TC9500 to control the temperature.

### 3.2. The Treatment Outcomes Obtained Under Stringent Ex Vivo Conditions Have High In Vivo Relevance

Under the stringent ex vivo model, both Silvadene and Flammacerium exhibited similar efficacy against *P. aeruginosa*, *K. pneumoniae*, *S. aureus*, *A. baumannii*, and *S. pyogenes*. This could potentially be due to the presence of Silver Sulfadiazine in both antimicrobial creams. However, Flammacerium also contains cerium nitrate, an antimicrobial agent that could enhance the antimicrobial activity of Silver Sulfadiazine [34], which might explain why Flammacerium showed higher efficacy than Silvadene against *E. faecium*.

The overall treatment outcomes obtained from our ex vivo model are comparable to those published in vivo data. For example, 1% Silvadene treatment resulted in an approximately 2.7-log reduction in the *A. baumannii* viable count in our ex vivo model (Figure 4) that is highly comparable to the 3-log reduction in *A. baumannii* burden by Silvadene obtained from a murine model of wound infection, a study conducted by Blanchard, C., et al. [35].

Additionally, our ex vivo model showed that Sulfamylon was more effective than Silver Sulfadiazine at reducing *Pseudomonas* CFU counts. Similar outcomes were observed in an in vivo study conducted by Murphy, R.C., et al. [36], in which mafenide acetate (either in cream or in solution) showed greater antimicrobial activity than Silvadene (Silver Sulfadiazine) in *Pseudomonas*-infected rat burn wounds. In the current study, the treatment effects of Flammacerium against *Pseudomonas* were comparable in both stringent ex vivo and in vivo (rat) testing conditions, resulting in similar log CFU reductions compared to the no-treatment controls (Figure 3). In contrast, the treatment outcomes of Silvadene and Flammacerium obtained from the conventional ex vivo model were significantly higher than those of the in vivo rat study (Figure 3).

Furthermore, in our ex vivo model, we showed that Mupirocin cream was more effective at reducing the *S*. *aureus* (MRSA) burden in the infected burn wounds than Silvadene and Flammacerium. Consistent with our ex vivo results, Rode et al. showed that a one-time Mupirocin treatment reduces MRSA by about 2 log (from approximately 1.85 × 10^8^ to 3.21 × 10^6^ CFU/g of tissue) in rat full-thickness burn wounds [37]. A similar trend was observed also in a clinical study conducted in South Africa, showing that MRSA infections that could not be controlled by 1% Silvadene were eliminated by Mupirocin treatment [38].

Although Silvadene is the leading topical therapeutic for controlling burn wound infections, our stringent ex vivo outcomes indicate that its efficacy against commonly isolated burn pathogens was limited. Among the six tested pathogens, none of the Silvadene treatments resulted in greater than a 3 log reduction in CFU counts. The outcomes from many clinical studies have raised questions regarding the effectiveness of Silvadene treatment for burn wound infections [39,40,41]. Continuous search for better topical product(s) to control burn infections is warranted. Our ex vivo model could be a valuable tool for screening and down-selecting topical agent(s) for effective control of burn infections before transitioning into biofilm-mediated infection models or animal studies.

### 3.3. Limitations of Stringent Ex Vivo Model

Although our ex vivo model acts as an excellent platform for screening antimicrobials, it does have some limitations, such as the lack of blood circulation and an immune system to recapitulate the host environment fully. The model also does not represent chronic wounds or biofilm-mediated wound infections. However, the current ex vivo model could be adapted for studying biofilm-mediated wound infections by lengthening the duration of incubation after inoculation and before treatment to allow biofilm formation to occur on the skin wounds. 

## 4. Materials and Methods

### 4.1. Bacterial Strains and Growth Conditions

*P. aeruginosa* strain 1244, *K. pneumoniae* BAMC 07-18, *A. baumannii* WRAMC#13, and Vancomycin-Resistant *Enterococcus faecium* WRAMC#3 clinical isolates were obtained from the U.S. Army Medical Centers. *S. aureus* TCH1516 (MRSA strain) and *S. pyogenes* ATCC 19615 were purchased from the American Type Culture Collection (ATCC). Bacteria were cultured in Tryptone Soy Broth (TSB) at 37 °C with or without CO_2_ based on the growth requirements of each bacterial strain and were grown to the mid-log growth phase for the inoculum.

### 4.2. Chemicals and Materials

Porcine skin sheets were purchased from Pel-freez Biologicals (Rogers, AR, USA). Skins were individually vacuum sealed and stored at −80 °C upon receipt for future use. Betadine was obtained from Avrio Health L.P. (Stamford, CT, USA). The commercial sources of the treatment agents were as follows: Mupirocin cream: Encube Ethicals, Inc., Durham, NC, USA; Sulfamylon cream: Rising Pharma Holdings, Inc., East Brunswick, NJ, USA; Silvadene cream: Greenstone LLC., Peapack, NJ, USA; and Flammacerium cream: Alliance Pharma Ltd., Dublin, Ireland. All chemicals, reagents, and growth media used in this study were obtained either from Fisher Scientific (Chicago, IL, USA) or Sigma (St. Louis, MO, USA).

Selective plates were purchased from various sources. *Pseudomonas* isolation agar plates: TEKNOVA, Hollister, CA, USA; Leeds *Acinetobacter* Medium and HardyCHROM^TM^ MRSA: Hardy Diagnostics, Springboro, OH, USA; *Klebsiella* ChromoSelect Agar: Millipore, Milwaukee, WI, USA; *Streptococcus* Selective agar and SPECTRA VRE: Remel, Lenexa, KS, USA; Sheep blood (5%) TSA agar plates and CHROMagar^TM^ *S. aureus* selective plates: Becton, Dickinson and Company, Sparks, MD, USA.

**Figure 5:**
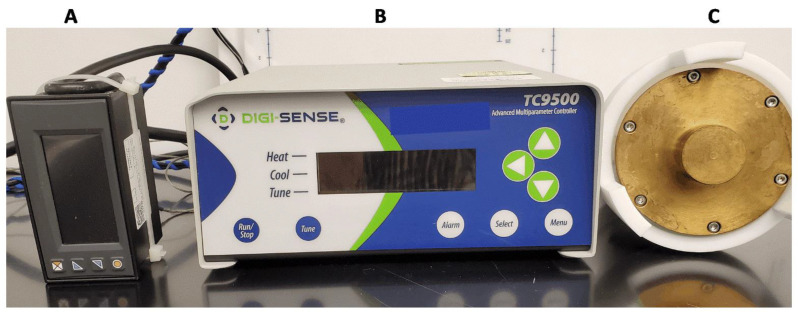
The burn device used in the ex vivo procedure. (**A**) pressure gauge, (**B**) Multiparameter controller, and (**C**) burn block showing the 3 cm-diameter cylinder.

The burn device was designed in our laboratory by Dr. Tao You and built by a local machine shop. The burn device was connected to a pressure gauge DP400S from Omega Engineering (Irlam, Manchester, UK) to monitor the applied pressure and to the Advanced Multiparameter controller TC9500 from Cole Palmer (Vernon Hills, IL, USA), to control the temperature of the burn block (Figure 5).

### 4.3. Ex Vivo and In Vivo Procedures

**Figure 6:**
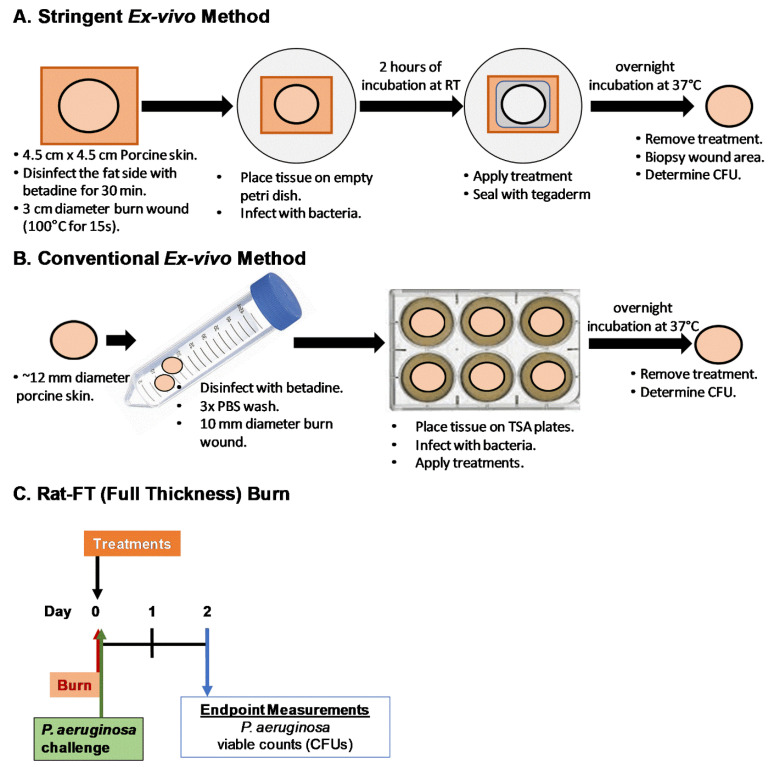
Schematic diagrams of different experimental designs used in this study, (**A**) Stringent ex vivo, (**B**) Conventional ex vivo, and (**C**) Rat-FT (Full-thickness) burn wound model using Silvadene and Flammacerium cream as treatments.

The experimental procedures are shown in the schematic diagrams below (Figure 6).

#### 4.3.1. Stringent Ex Vivo Screening Model

Porcine skins were thawed on ice or at 4 °C for two hours before use. Thawed skins were cut into approximately 4.5 cm × 4.5 cm squares. In in vivo scenarios, the commensals are present on the surface of the skin and there are no bacteria under the bottom of the skin. Therefore, to preserve the skin surface commensals and to minimize bacterial contamination under the skin during the tissue collection processing, skin pieces were placed on a petri dish (150 × 25 mm) with a 100 × 100 mm non-woven, 4 ply gauze underneath and treated with approximately 30 mL of betadine for 30 min, making sure that only the bottom layer was submerged in betadine and the skin surface was untouched. Carefully, the skins were then washed in PBS (3×, 10 min/wash) to remove betadine residues and blot-dried with dry gauze. A 3 cm-diameter thermal injury was created on each square of porcine skin using the burn device with the temperature set at 100 °C for a burn duration of 15 s. The burned skin was then placed on a new petri dish, and the burn area was inoculated with 20 µL of approximately 1 × 10^6^ CFU/mL of the test pathogen. After a 2-h incubation at room temperature, the burn wounds were treated with 1 mL of Silvadene cream, Flammacerium cream, Mupirocin cream, or Sulfamylon cream, covered with Tegaderm, and incubated at 37 °C overnight with or without CO_2_ based on the growth requirements of the test pathogen(s). After incubation, the topical treatments were removed, the burned skin was punched out, placed in a 50 mL Bigprep Lysing Matrix D tube (MP Biomedicals, Solon, OH, USA) containing 25 mL of D/E Neutralizing Broth (Remel, Lenexa, KS, USA), and homogenized using a tissue homogenizer (MP Biomedicals, Solon, OH, USA). The homogenates were serially diluted, plated on pathogen-specific selective plates, and incubated with or without CO_2_ at 37 °C for approximately 20 h. After incubation, the number of colonies on the plates was quantified using Protocol3 (Synbiosis, Microbiology International, Frederick, MD, USA), and the CFU/wound was calculated. The treatment effect was assessed by comparing the viable counts of the test pathogen recovered from the treated vs. the untreated samples.

#### 4.3.2. Conventional Ex Vivo Model

The ex vivo method was adopted and modified from the literature [14,20]. After the porcine skin was thawed, 12 mm diameter circular pieces were cut, and about 15 biopsy punches at a time were placed in a 50 mL conical tube with 25 mL betadine for disinfection of the skin. The skins were then washed 3× with PBS and blot-dried with sterile gauze. A 10 mm diameter burn was created on each skin piece (100 °C for 15 s) using a burn device with a 10 mm burn block. The burned skin piece was placed on top of a TSA plate, and the burn area was inoculated with 10 µL of approximately 1 × 10^6^ CFU/mL of *P. aeruginosa,* and the wound area was treated with 100 µL of either Silvadene or Flammacerium cream. After overnight incubation at 37 °C, the skin was homogenized, processed, and plated on *Pseudomonas* selective plates. Viable *P. aeruginosa* CFUs were counted, and the treatment efficacy was calculated, as described in Section 4.3.1.

#### 4.3.3. Rat Model of Burn Wound Infection

Animal Ethics Statement: Research was conducted in compliance with the Animal Welfare Act, the implementing Animal Welfare regulations, and the principles of the Guide for the Care and Use of Laboratory Animals. The Institutional Animal Care and Use Committee approved all research conducted in this study. The facility where this research was conducted is fully accredited by AAALAC International. The in vivo treatment procedures were performed as described in previous studies [42,43]. Briefly, 27 male Sprague-Dawley rats (9 rats per group) in the weight range of 350 to 450 g were used in the study (part of the approved U.S. Army Institute of Surgical Research IACUC protocol A-16-020). The day before the scalding, the dorsum side of the rats was shaved and depilated with Nair under anesthesia using 2.5–4% isoflurane (Forane, Baxter Healthcare Corporation, Deerfield, IL, USA) and Buprenorphine SR LAB (1.2 mg/kg; Zoopharm Pharmacy, Laramie, WY, USA) was administered subcutaneously for proactive pain management. The experimental design is shown in Figure 6C. Briefly, approximately 10% TBSA full-thickness burns were created on the shaved dorsum of the rat by exposing it to 99–100 °C water for 6 sec in a Precision CIR 35 circulating water bath (Thermo Scientific, Waltham, MA, USA). After the rats were photographed and administered resuscitation fluid, the top of the burn wound was inoculated with 100 µL (approximately 1 × 10^6^ CFU per mL) of *P. aeruginosa.* The inoculum was spread across the whole wound using a pipette tip. Immediately post-infection, approximately 5 mL of treatment cream was applied once, and the wound was covered first with a non-adherent dressing, the N-terface (Winfield Laboratories, Inc., Richardson, TX, USA), and then with the Tegaderm Film (3M Health Care, St. Paul, MN, USA). The edges of the Tegaderm were sealed with NOTAPE professional silicone bonding adhesive (Vapon, Inc. Fairfield, NJ, USA).

On day 2, rats were anesthetized with 100 mg/kg Ketamine HCl and 10 mg/kg Xylazine. Euthanasia was achieved by direct intra-cardiac injection of Fatal-Plus^®^ (Vortech Pharmaceuticals, Dearborn, MI, USA) and confirmed by lack of cardiac movement, pulse, and breath. Upon confirming the death, the entire burn area was excised, and biopsy punches (7 mm) were taken from the burn area for bacterial quantification as described previously [42]. The treatment effect was graphed in log scale.

### 4.4. Statistical Analysis 

GraphPad Prism 10.2.0 (GraphPad Software, Inc., San Diego, CA, USA) was used to plot and analyze the data. Groups were compared using one-way ANOVA and two-way ANOVA (with Tukey post-hoc test). *p*-values below 0.05 were considered significant.

## 5. Conclusions

We have developed a stringent ex vivo model of burned porcine skin wound infections to bridge the gap between the in vitro and in vivo models for screening the antimicrobial activity of various topical antimicrobial agents and formulations. We demonstrated that outcomes obtained from our ex vivo model have high in vivo relevance, and this could increase success rates in subsequent animal studies and, therefore, reduce animal usage and overall cost.

## Data Availability

The original contributions presented in the study are included in the article, further inquiries can be directed to the corresponding author.
